# Radiative cooling textiles using industry-standard particle-free nonporous micro-structured fibers

**DOI:** 10.1515/nanoph-2023-0650

**Published:** 2024-01-11

**Authors:** Peter B. Catrysse, Shanhui Fan

**Affiliations:** E. L. Ginzton Laboratory and Department of Electrical Engineering, Stanford University, Stanford, CA 94305, USA.

**Keywords:** radiative cooling, textiles, micro-structured fibers, nonporous, particle-free, industry-standard

## Abstract

Thermal radiation is a major dissipative pathway for heat generated by the human body and offers a significant thermoregulation mechanism over a wide range of conditions. We could use this in garment design to enhance personal cooling, which can improve the wearing comfort of garments or even result in energy savings in buildings. At present, however, radiative cooling has received insufficient attention in commercial design and production of textiles for wearable garments. Textiles that efficiently transmit the radiative heat were recently demonstrated, but either do not utilize standard weaving and knitting processes for wearable garments or require substantial process modifications. Here, we demonstrate the design and implementation of large-scale radiative cooling textiles for localized cooling management and enhanced thermal comfort using industry-standard particle-free nonporous micro-structured fibers that are fully compatible with standard textile materials and production methods. The micro-structured fibers, yarns and fabrics are part of a hierarchical photonic structure design that renders the textiles highly infrared transparent (up to > 0.8) while assuring visual opacity (up to 0.99). We design radiative cooling textiles with first-principles electromagnetic methods and fabricate them using commercial textile materials and formation facilities. Our “fabless” approach is confirmed by very good quantitative agreement between design and measurements. The resulting fabrics exhibit wearability properties expected of wearable textiles, and lower skin temperature by ≥ 3 °C compared to conventional textiles, which offers the potential for > 30 % energy savings in buildings and increases wearing comfort by significantly reducing the reliance on latent heat dissipation for thermoregulation.

## 1 Introduction

Thermal radiation is a major pathway for dissipating heat generated by the human body to the ambient environment. It applies very generally to both indoor and outdoor environments if the ambient is at a lower temperature than the skin. For indoors, in particular, radiative heat transfer contributes more than 65 % of total body heat loss during typical sedentary activities and remains the main heat transfer pathway at light to moderate activity levels [1]–[3]. Provided thermal radiation can dissipate away from the body efficiently, radiative heat transfer offers a significant thermoregulation mechanism over a wide range of conditions [1]. We can exploit this for localized thermal management in a purely passive way with garment design, which can result in energy savings for buildings, but it also creates an opportunity for enhancing personal cooling, which can improve the wearing comfort of garments [4]–[7].

Efficient transfer of radiative heat from the body to the environment, however, has hitherto received insufficient attention in the commercial design and production of textiles for wearable garments. Fabric formation for wearable garments relies on modern weaving and knitting methods [8], [9]. The woven and knitted fibers and yarns are typically made of materials, either natural (e.g., cotton) or synthetic (e.g., polyester), which are essentially opaque at thermal infrared (IR) wavelengths where the body emits radiative heat [10]. Hence, current commercial textiles made for wearable garments block the radiative cooling pathway. Instead, they facilitate thermoregulation of the human body by controlling the thermal heat flow to the environment using non-radiative pathways, such as convective, conductive, and latent heat transfer. The potential offered by the radiative heat transfer pathway has, therefore, largely remained untapped.

Recently, radiative cooling textile designs that efficiently transmit the thermal radiative heat emitted by the skin to the environment have been proposed and demonstrated using materials that are highly transparent at IR wavelengths [5], [6], [11], [12]. Since these initial demonstrations, there has been a large body of work on radiative cooling for textiles [13]–[15]. The majority of these works, however, either does not utilize standard weaving and knitting processes used in fabric formation methods for wearable garments [8], [9], or requires substantial modifications of such processes [14]. While there are published works on woven or knitted textiles, the approaches for achieving radiative cooling that have been shown to date are based on modifying fibers either by introducing (nano)pores into the fibers or by adding (nano)particles through embedding in or as coatings on the fibers [5], [7], [12], [16]–[18]. A design method and an implementation process to create radiative cooling textiles based on micro-structured fibers without pores or particles that relies on industry-standard textile fiber-grade materials and processing steps only, on existing industry-scale fiber/yarn production equipment and commercial fabric industry weaving or knitting tools, has not been shown yet.

Here, we demonstrate large-scale textiles for localized radiative cooling and enhanced thermal comfort using industry-standard particle-free nonporous micro-structured fibers that are fully compatible with standard textile materials and production processes. The micro-structured fibers, the yarns comprised of them and the fabrics formed with them are part of a hierarchical photonic structure design that renders the resulting textiles highly transparent in the thermal IR range while assuring opacity in the visible range. To implement these large-scale radiative cooling photonic structure textiles, we use only commercial fiber and yarn production tools, as well as textile industry fabric formation tools. Hence, the approach presented here is entirely implemented with existing commercial fiber and yarn fabrication processes, textile weaving and knitting methods, and is therefore industry-compatible. The resulting photonic structure textiles not only provide superior cooling performance, but also exhibit wearability properties expected of woven and knitted textiles.

## 2 Design, implementation, and characterization

To select materials for the design and implementation of radiative cooling textiles using particle-free nonporous micro-structured fibers, we consider both their radiative properties in the thermal IR range and their ability to be used in fiber and yarn production for fabric formation. For the demonstration in this work, we opt for polyethylene (PE) and polypropylene (PP). PE has only a few narrow absorption features in thermal IR range where the human body emits radiative heat and is highly transmissive at these wavelengths [19]. However, it is not commonly used as fiber material in textiles and it has a low melting point [20], [21], which may limit routine garment care (e.g., ironing, commercial drying). PP, on the other hand, is more absorbing at thermal IR wavelengths, but is more commonly used for textile fibers [9], [21]. To create radiative cooling textiles, we design particle-free nonporous fibers that combine both materials to achieve high IR transmissivity for excellent cooling performance.

We use first-principles electromagnetic (EM) simulations to assess a textile design by calculating optical performance, i.e., how transmissive in the IR range and opaque in the visible range the textile is. High IR transmissivity is a strong indicator of radiative cooling ability [5], [6], [11], [12]. To calculate visible and IR transmissivity spectra, we employ full-vector EM field simulations based on a rigorous coupled wave analysis (RCWA) method [22]. Once a design is implemented, we compare the calculated IR spectra with spectral measurements from hemispherical incident IR radiation. To do this, rather than calculating only normal incidence and assuming that the radiative textile behavior is isotropic, as is typically done, we calculate the full spectral, directional transmissivity $\tau \left(\theta ,\phi ,\lambda \right)$ to account for correct transmissivity values across the full hemisphere (angles of incidence *θ* = 0 → *π*/2 and *ϕ* = 0 → *π*/2) [6], [23]. We then perform a full angular integration of the spectral, directional transmissivity to obtain angularly integrated spectra that can be compared to the full hemispherical measured transmissivity spectrum.

To implement the radiative cooling textiles based on particle-free nonporous micro-structured fibers and experimentally demonstrate our approach, we use industry-scale textile fiber extrusion equipment (Hills, Inc.) to produce micro-structured fibers and yarns with a filament melt-spinning process [24]. Industry-standard fiber-grade polymer resins in pellet form suitable for industrial extrusion processes are used as source materials for the fibers and yarn. To show the robustness of our design to the choice of material source, we procured PP and PE resins from industrial manufacturers (Sunoco Chemicals, The Dow Chemical Company). The melt-spinning process produces multifilament yarn consisting of filament fiber bundles. We subsequently use this yarn in commercial fabric formation tools to create woven fabric samples on a rapier loom (CCI Tech Inc.) and knitted fabric samples on a knitting machine (Lawson Hemphill Inc.) in a “fabless” manner.

We determine thermal transmissivity and visual opacity experimentally by characterizing the optical properties of the radiative cooling textiles based on particle-free nonporous micro-structured fibers in the IR and visible ranges. The spectral transmissivity $\tau \left(\lambda \right)$ in the IR range is measured using Fourier-transform infrared spectroscopy (FTIR) with a spectrometer (Thermo Scientific, Model 6700) and a diffuse gold integrating sphere (PIKE Technologies). The specular transmissivity in the visible range is measured using a UV-visible spectrometer (Agilent, Cary 6000i). The visible opacity was determined as (1 – specular transmissivity) [5].

To measure the cooling performance of radiative cooling textiles based on particle-free nonporous micro-structured fibers directly, we use a steady-state, constant heat flux thermal setup. The skin is simulated using an 8 cm × 8 cm silicone rubber electric heater (Omega), which is connected to a power supply and which has a thermal IR emissivity similar to that of human skin [7]. A k-type thermocouple (Omega) is attached on the center of the top surface to measure skin temperature *T*_*skin*_. An 8 cm × 8 cm guard heater is placed below the skin with a k-type thermocouple attached on the center of the bottom surface to measure *T*_*guard*_, which is always kept the same as *T*_*skin*_ to ensure one-directional heat flux *q* towards the textiles. The skin and the tested sample are placed in an enclosure that is kept at constant air temperature *T*_*amb*_. For all thermal measurements *T*_*amb*_ = 23.3 °C and the power supply is set to provide a constant flux *q* = 89 W/m^2^. When different textile samples (size 5 cm × 5 cm) are placed onto the skin, *T*_*skin*_ will change accordingly.

We assess the wearing comfort of the radiative cooling textiles based on particle-free nonporous micro-structured fibers and yarns by measuring important wearability properties, such as water wicking rate, air permeability, and water vapor transmission rate. The wicking test procedure follows the AATCC TM 197 specification. The air permeability test procedure follows the ASTM D737 specification. The water vapor transmission rate test follows the ASTM E96 specification.

## 3 Results

**Figure 1:**
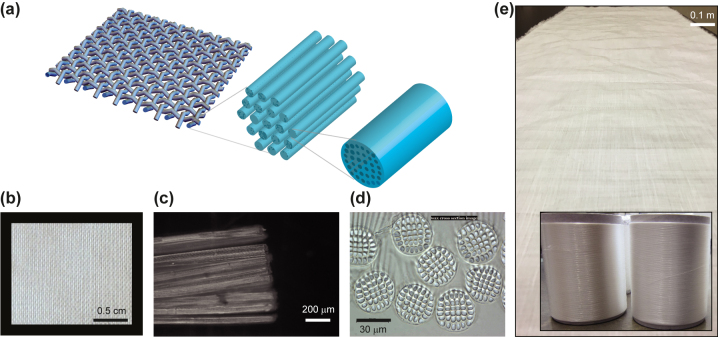
Fiber, yarn, and fabric design and production process for radiative cooling textiles based on industry-standard particle-free nonporous micro-structured fibers. (a) Multi-scale design of radiative cooling textile design based on particle-free nonporous micro-structured fibers. Shown are the different components and scales of the design as represented by the woven or knitted fabric made of yarn, where each yarn is a bundle composed of micro-structured fibers, in which each fiber is micro-structured with an array of parallel fibrils embedded in its core, (b) close-up photograph of the woven fabric, (c) microscope image of the yarn composed of micro-structured fiber bundle, (d) optical microscope image of the particle-free nonporous micro-structured fibers, (e) large-area woven fabric sample made on an industry-standard loom with inset showing large spools of yarn made using industry-scale yarn production tools.

**Figure 2:**
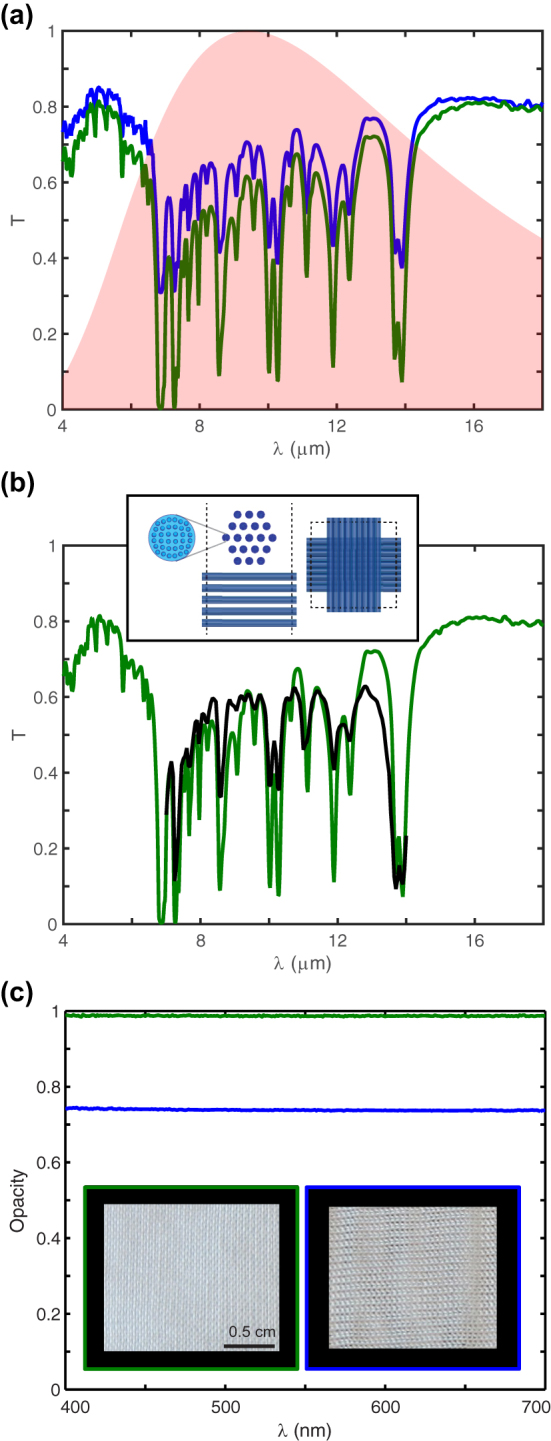
Optical characterization of fabrics based on industry-standard particle-free nonporous micro-structured fibers: (a, b) IR optical characterization of knitted and woven textiles. The FTIR measured transmissivity is shown in panel (a) for knitted (blue) and woven (green) textile samples, which are shown in the inset of panel (c). Panel (b) shows the calculated IR transmissivity of woven textiles. The graph shows FTIR measured (solid green line) and RCWA calculated (solid black line) transmissivity for the woven textile. Inset shows the radiative cooling textile model. Cross-sectional geometry shows one period of the periodic arrangement of yarn (side view left, top view right), in which the yarn are crossed and vertically offset with respect to each other to model the woven nature of the textile. Each yarn consists of an array of closely packed particle-free nonporous micro-structured fibers. (c) Visible optical characterization of knitted and woven textiles. The graph shows visual opacity for knitted (blue) and woven (green) textiles shown in the inset.

Figure 1 presents the design and the production process of radiative cooling textiles based on industry-standard particle-free nonporous micro-structured fibers and yarns. We employ a holistic multi-scale approach to the design and the implementation of radiative cooling textiles. This process starts with the photonic design of particle-free nonporous micro-structured fibers and ends with the formation of large-area woven and knitted radiative cooling textiles. We use a hierarchical photonic approach that includes design of fibers, yarn based on these fibers, and the woven or knitted fabric. Figure 1a illustrates the different components and scales of the design. The fabric design geometry is formed by a periodic arrangement of crossed yarn, corresponding to weft and warp orientation in which weft and warp yarn are vertically offset with respect to each other to model the woven nature of the textile (Figure 2b, inset). Each yarn consists of a (lengthwise) parallel array of fibers forming a closely packed bundle. Each fiber in this bundle is a particle-free nonporous micro-structured fiber.

The micro-structured fiber geometry is formed by embedding a parallel array of fibrils inside the fiber core. The fibrils differ in material from the fiber core and are micrometer scale. Hence, they are sub-wavelength for IR wavelengths, which limits the scattering of thermal radiation to maintain high IR transmissivity [5], [6], [11]. They are spaced by distances comparable to visible wavelengths, which enables scattering in the visible range to create opacity [5], [6], [11].

For the implementation of the design, we use industry-standard textile processes only. Figure 1b shows a close-up image of a woven fabric (∼300 μm periodicity) on a black background to demonstrate visual opacity. Figure 1c is a microscope image of the yarn used to produce this fabric. It shows that each yarn is a multi-fiber bundle with a diameter of ∼200 μm. Figure 1d shows a microscope image of individual particle-free nonporous micro-structured fibers that make up the yarn. Each fiber is micro-structured with fibrils embedded in the fiber body that extend along the full length of the fibers. Each individual micro-structured fiber has a diameter of ∼30 μm, while the fibrils inside each fiber measure ∼5 μm in diameter. Figure 1e shows a large-area sample of fabric formed with yarn based on the micro-structured fibers on an industry-standard rapier loom. The length scale of the fabric in width is meter size with a length limited only by the yarn supply. The samples have a thickness of ∼350 μm (knitted) and ∼200 μm (woven), which is comparable to fabrics used for wearable garments. The inset shows large spools of yarn with the micro-structured fibers, which are made on industry-standard melt-spinning production tools and form the supply for the industry-scale fabric formation.

The formation of radiative cooling textile, as shown in Figure 1, is based on a production process that uses only commercial textile fabric formation prototyping tools and serves to demonstrate that the particle-free nonporous micro-structured fiber approach is entirely compatible with existing industry-standard yarn production and fabric formation tools. We emphasize that the production process requires only existing fiber and yarn materials. Unlike previous approaches based on nanoporous or nanoparticle based fibers, it does not rely on the development, introduction and/or adoption of new materials. This can be an advantage in an industry based on low-cost, large-scale volume production, in which the introduction of new materials can be challenging.

We perform an optical characterization of the radiative cooling textiles based on industry-standard particle-free nonporous micro-structured fibers in the thermal IR and visible ranges. Figure 2a shows IR transmissivity spectra for knitted (solid blue) and woven (solid green) fabric samples. The transmissivity for knitted and woven fabrics is large in the 4–18 μm thermal IR range, which overlaps with the radiative heat spectrum of the human body (shown as a background in the graph). The spectra feature several dips. The dips near 7 and 14 μm correspond to absorption features of PE, i.e., C–C bending at 6.8 μm, C–C wagging at 7.3 μm, and C–C rocking at 13.7 μm, respectively [19]. The spectral dips between 8 and 13 μm are due to absorption features of PP at 10.2, 11.1, 11.8, and 12.3 μm (C–C rocking), respectively. While the locations of the spectral dips correspond to spectral signatures of the materials used in the design, the measured transmissivity values at these wavelengths depend on the actual geometry of the design. As a result, we find that the transmissivity in the thermal IR range reaches above 0.8 for both fabrics with averages of 0.70 and 0.59 for knitted and woven fabric samples, respectively.

We then compare the optical measurements with first-principles EM calculations of the radiative cooling fabric design. We use design parameters for the fiber diameter (30 μm), diameter of the embedded fibrils (4.5 μm), gap between embedded fibrils (0.5 μm), gap between the 19 filament fibers of each yarn (5 μm), and gap between crossed yarns (35 μm), rather than measured values for the actual radiative cooling textile samples. We also derive the yarn periodicity (290 μm) from the weft and warp design settings in the fabric formation process (accounting for crimp), rather than actual measured values. The use of design parameters in the calculations when comparing to the optical measurements serves to show the robustness of our multi-scale fabric design to variations in the parameter values as will be encountered in commercial yarn fabrication and fabric formation processes. Figure 2b shows the calculated thermal IR transmissivity of a woven radiative cooling fabric. The calculated (solid black) transmissivity spectra fit the measured spectra (solid green) very well. There is very good overall quantitative agreement as well as matching absorption dips in location and strength. This demonstrates the ability to design radiative cooling textiles and to fabricate them in a “fabless” fashion using only commercial textile materials and formation facilities, which are not under our control, and yet be able to obtain very good quantitative agreement between the design and the measured optical properties.

To evaluate the effect of different design parameters on the transmissivity of the fabric, we performed additional simulations for a range of parameter values. Regarding the size of the fibrils, we find that a change of up to 20 % in fibril diameter impacts transmissivity by less than 6 %. The finding that changing fibril size does not affect IR transmissivity significantly is expected since their sub-wavelength size limits scattering of thermal radiation. On the other hand, changing the yarn periodicity can have a significant impact. When varying yarn periodicity between 290 μm and 430 μm, we find a 28 % improvement in transmissivity with reached values increasing from 0.63 to 0.74. By also varying the fiber diameter from 30 μm to 18 μm, the transmissivity can be further increased and reaches values up to 0.79. While we already showed high IR transmissivities for the design we implemented, these results suggest that there exist possibilities to improve performance further. Moreover, while these are results for a design that already includes a more absorbing material, the ability to reach even higher levels of transmissivity by geometry design also points to an opportunity to include even less IR transparent textile materials.

The results for the optical characterization in the visible range are shown in Figure 2c. Visual opacity is the important property of textiles in the visible wavelength range (400–700 nm). It represents the ability to prevent objects behind the textile (e.g., skin) to be visible from the outside. Visual opacity is quantified as (1 – specular transmissivity) [5]. Figure 2c shows that the opacity of the knitted and woven fabrics is 0.75 and 0.99, respectively, over the entire visible range. By comparison, cotton has an opacity of 0.99. The opacity remains at this level for wavelengths smaller than 400 nm, which means that the woven fabric in particular also exhibit ultraviolet opacity and can protect the skin in this wavelength range. We also visually observe the opacity of the knitted and woven radiative cooling textiles in the insets of Figure 2c. Both the woven and knitted fabrics based on particle-free nonporous micro-structured fibers are visibly white and opaque. This is attributed to the scattering of visible light when interacting with the multi-scale fabric design, which comprises contributions from the micro-structured fibers, the multi-fiber bundle yarns, and the woven or knitted fabric structure. Hence, the response in the visible range is strongly dependent on the design geometry. First-principles EM field simulations of the fabric design model (inset of Figure 2b), performed at visible wavelengths, confirm this and yield a visual opacity of 0.98, which closely matches the experimentally measured opacity of the woven fabric (0.99). This also again showcases the robustness of our fabless design approach.

The optical characterization of the knitted and woven fabrics provides strong indirect evidence for their radiative cooling ability. We confirm their cooling performance directly with thermal measurements. To quantify cooling performance, we use a thermal setup that measures skin temperature when covered with a radiative cooling fabric and we compare this to the skin temperature of bare skin (without fabric covering) and when skin is covered with a conventional cotton textile.

**Figure 3:**
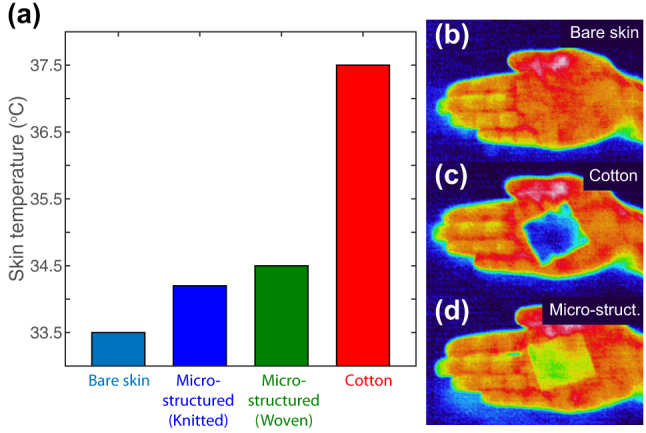
Thermal characterization of radiative cooling fabrics based on particle-free nonporous micro-structured fibers: (a) Skin temperature for bare skin, for skin covered with knitted and woven textiles based on micro-structured fibers and for skin covered with conventional cotton textile. Thermal IR images of (b) uncovered hand (bare skin), (c) cotton textile and (d) woven fabric based on particle-free nonporous micro-structured fibers just after they have been placed on skin of hand and while they have not reached thermal equilibrium with the skin yet.

Figure 3a presents thermal characterization measurements. The skin temperature of bare skin (33.5 °C) is shown as reference. The knitted and woven fabrics based on micro-structured fibers and yarns result in skin temperatures of 34.2 °C and 34.5 °C, respectively. This is only 0.7 °C and 1 °C above the bare skin temperature. It is also 3.3 °C and 3 °C lower, respectively, than the skin temperature for the conventional textile based on cotton (37.5 °C). This demonstrates that woven and knitted fabrics based on industry-standard particle-free nonporous micro-structured fibers provide significant cooling and can achieve temperatures close to bare skin. To put this cooling performance in context, woven and knitted cooling textiles based on nanoporous fibers have been shown to lower skin temperature by 2.3 °C as compared to conventional cotton textiles [12]. In addition to thermal setup results, we use thermal imaging to visualize the radiative cooling behavior. Figure 3b shows a thermal IR image of an uncovered hand. Figure 3c and d depict thermal images of a cotton fabric (c) versus our woven fabric based on particle-free nonporous micro-structured fibers and yarns (d) shortly after they have been placed on top of human skin. The fabrics have not reached thermal equilibrium with the skin yet. Their temperature is therefore still at or close to the ambient temperature (dark, blue color of background). The dark, blue color for the cotton fabric in the top image demonstrates that the radiative thermal emission from the skin is blocked by the cotton. The woven radiative cooling fabric, on the other hand, is a bright, yellow color and demonstrates that it largely transmits the thermal emission from the underlying skin.

The temperature measurements for radiative cooling fabrics based on particle-free nonporous micro-structured fibers demonstrate ≥ 3 °C reduction in skin temperature, when compared to 37.5 °C for a conventional cotton textile. Given that the differences between the skin and the ambient temperatures (23.5 °C) are small, these skin temperature differences also approximate the air conditioner set-point differences [5], [12].

Hence, a decrease in skin temperature, as shown, corresponds to an equal set-point increase for building temperatures [12], and a set-point increase of ≥ 3 °C can result in more than 30 % energy savings on the cooling of buildings [4], [6], [12]. We find therefore that the use of particle-free nonporous micro-structured fibers can result in significant energy savings for the cooling of buildings.

Beyond energy savings in buildings, radiative cooling textiles based on particle-free nonporous micro-structured fibers can also significantly impact the wearing comfort of textiles in general. When the body runs out of sensible thermoregulation options, it has to resort to latent heat dissipation, i.e., we start to perspire or sweat, which causes discomfort. To mitigate any discomfort from perspiration, conventional textiles are engineered for wicking to remove sweat from the body through the textile [25]. Reliance on this latent heat dissipation may be reduced or even eliminated in many circumstances and the wearing comfort can thus be increased, provided that textiles are designed to allow effective radiative heat transfer. The effect in this broader comfort context is two-fold. First, radiative cooling textiles based on particle-free nonporous micro-structured fibers can delay the onset of sweating. The onset of sweating has been shown to correspond to a skin temperature above 34.5–35 °C for light to moderate effort [26], which is above the measured skin temperature for our textile samples. Next, when sweating starts, they can lower the sweating rate significantly. A decrease in skin temperature by more than 2 °C, as our radiative cooling textiles demonstrate, has been shown to result in a more than 5-fold reduction of the sweating rate once sweating starts [26], [27].

**Figure 4:**
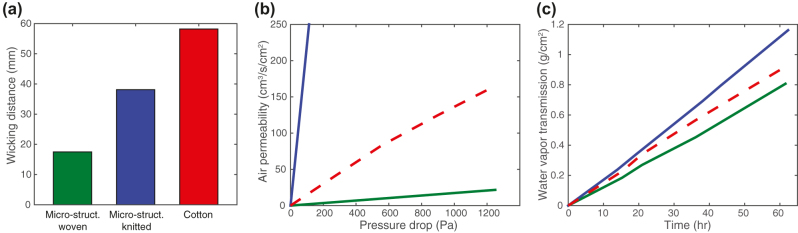
Wearability test results for woven and knitted radiative cooling textiles based on particle-free nonporous micro-structured fibers. (a) Wicking distance test shows the ability to transport perspiration for quick evaporation. (b) Air permeability test examines the air flow rate through the textile at certain pressure drops. (c) Water vapor transmission rate test shows how human perspiration can transmit through the textile.

While efficient radiative heat transfer ensures the cooling performance of radiative cooling fabrics based on particle-free nonporous micro-structured fibers, we also assess their wearing comfort by measuring important wearability properties. The results of these industry-standard textile characterizations are shown in Figure 4. Figure 4a shows wicking rate, which measures the ability to transport perspiration for quick evaporation to avoid any discomfort. We compare our textiles to cotton, which is known for a large wicking rate due to its hydrophilic cellulose fibers. The micro-structured fiber fabrics also show reasonable wicking rates of 17.5 mm (woven) and 38.1 mm (knitted) in 60 s. This can be attributed to the design of the yarn design with small fiber spaces in the filament bundle [28], [29]. The air permeability represents the ability of the textile to allow air to flow through, which facilitates heat transfer through convection (Figure 4b). Using a standardized measurement, we find an air permeability of 0.13 and 16 l/min/cm^2^ for the woven and knitted textiles, respectively. These are within the suitable range of air permeability for textiles and comparable with a measurement of 0.9 l/min/cm^2^ for cotton [30], [31]. The water vapor transmission rate is an indicator for how human perspiration is transmitted through the textile (Figure 4c), which is important to avoid discomfort of wet clothing. The water vapor transmission rate of the woven (green curve) and knitted textile (blue curve) is 125 and 184 g/m^2^/h, respectively, which is comparable to the rate of 155 g/m^2^/h for cotton (red curve).

## 4 Discussion

We have demonstrated large-scale woven and knitted fabric textiles for localized radiative cooling and enhanced thermal comfort using industry-standard particle-free nonporous micro-structured fibers. The textiles are based on a hierarchical photonic structure design that includes micro-structured fibers, yarns made of fiber bundles, and woven and knitted fabrics formed with the yarns. We used first-principles EM field simulations to design textiles that are highly transparent in the thermal IR range and opaque in the visible range. The textile designs were then made in a fabless manner on commercial fabric formation tools used in the textile industry, with yarn made from micro-structured fiber bundles produced on industry-scale extruders using industry-standard fiber materials only. Hence, the approach presented here is entirely implemented with existing commercial fiber and yarn fabrication processes, textile weaving and knitting methods, and is therefore industry-compatible. Optical measurements of the resulting textiles at infrared and visible wavelengths show very good quantitative agreement with the photonic structure design, which validates the “fabless” approach. We found that radiative cooling textiles based on particle-free nonporous micro-structured fibers made using industry-standard fiber production and fabric formation tools can achieve both high transparency (up to > 0.8) in the thermal infrared range as well as very high visual opacity (up to 0.99), which rivals the opacity of conventional textiles. Thermal measurements show that these textiles can lower skin temperature by as much as 3.3 °C compared to conventional textiles. To contextualize these results, woven and knitted textiles based on nanoporous fibers have been shown to decrease skin temperature by 2.3 °C. By lowering the skin temperature by ≥ 3 °C, radiative cooling textiles based on particle-free nonporous micro-structured fibers can allow an increase in a building’s air conditioning set-point by the same amount, which offers the potential for more than 30 % energy savings in buildings. Moreover, latent heat dissipation may be reduced significantly or even eliminated when skin temperature can be lowered to ∼34.5 °C. With fabric thicknesses comparable to conventional fabrics and wearability properties expected of woven or knitted fabrics, wearing comfort in general can be increased as well. Since the implementations of the radiative cooling textiles were carried out with commercial yarn production and textile industry fabric formation tools only, the entire approach is inherently compatible with existing commercial fiber/yarn fabrication processes and textile weaving/knitting methods at industry scale.
